# Investigating the Interplay between Tomato Leaf Curl New Delhi Virus Infection, Starch Metabolism and Antioxidant Defence System in Potato (*Solanum tuberosum* L.)

**DOI:** 10.3390/antiox12071447

**Published:** 2023-07-18

**Authors:** Ravinder Kumar, Milan Kumar Lal, Rahul Kumar Tiwari, Kumar Nishant Chourasia, Awadhesh Kumar, Rakesh Kumar, Shivangi Sharma, Brajesh Singh

**Affiliations:** 1 ICAR-Central Potato Research Institute, Shimla 171001, Himachal Pradesh, India; chauhanravinder97@gmail.com (R.K.); dhimanrk16@gmail.com (R.K.); shivangisharmaangirous@gmail.com (S.S.); birju16@gmail.com (B.S.); 2 ICAR-Central Research Institute for Jute and Allied Fibres, Barrackpore 700121, West Bengal, India; chourasiakn@gmail.com; 3 ICAR-National Rice Research Institute, Cuttack 753006, Odisha, India; awadhesh.kumar@icar.gov.in

**Keywords:** starch metabolism, antioxidant activity, potato, ToLCNDV, source-sink

## Abstract

The potato apical leaf curl disease is caused by tomato leaf curl New Delhi virus-potato (ToLCNDV-potato), which severely alters a plant’s starch metabolism, starch hydrolysing enzymes, and antioxidant mechanism. In this study, the result suggested that ToLCNDV-potato significantly (*p* < 0.01) affected the morphological parameters and photosynthetic pigment system in both the cultivars of potato, viz., Kufri Pukhraj (susceptible) and Kufri Bahar (tolerant). However, the impact of ToLCNDV-potato was lower in Kufri Bahar. Moreover, the viral infection in potato showed significant (*p* < 0.01) enhancement in the leakage of plant oxidative metabolites such as proline and malondialdehyde (MDA) which was further confirmed with higher electrolyte leakage. The viral infection imbalance of starch metabolism in the leaves ultimately affects the carbohydrate profile. ToLCNDV-potato significantly lowered starch synthesis, enhanced the accumulation of sucrose, glucose, fructose and—which was further validated by enzymatic estimation of β-amylase—α-amylase and phosphorylase activity in the leaves of both cultivars. The antioxidant enzymes, viz., catalase, ascorbate peroxidase, and superoxide dismutase, were reported to be enhanced in both the cultivars due to ToLCNDV-potato infection. The higher enhancement of antioxidant enzyme activity was observed in Kufri Bahar, which signifies its resistant attributes. These findings in the potato plant broaden our understanding of the regulatory mechanisms of starch metabolism and antioxidant activity and provide proof of concept for breeding potato for ToLCNDV-potato tolerance.

## 1. Introduction

Potato (*Solanum tuberosum* L.) is an important solanaceous crop consumed globally after rice, wheat, and maize [1]. The cultivation of potato is widely spread in temperate and subtropical regions, as well as in various agroclimatic zones. As a result, potato plants are subjected to various biotic and abiotic factors that affect growth, development, and quality [1,2]. Biotic factors such as fungi, viruses, bacteria, and viroid infection significantly affect the metabolism of the potato plant and alter its physiological, biochemical and molecular responses against these stress factors. Earlier reports suggested that the pathogen infection negatively affects the potato plant’s morphological, physiological, and biochemical traits by altering its photo-assimilate distribution [3]. Among various pathogens affecting potato plants, viruses are important and crucial pathogens that pose the most detrimental effect on potato production worldwide and affect crop growth, development, production, and productivity [4]. Recently, tomato leaf curl New Delhi virus (ToLCNDV-potato) has been considered a devastating virus that significantly affects the potato plant that belongs to the genus Begomovirus in the family Geminiviridae [5,6]. More than 43 plant species are affected by the tomato leaf curl New Delhi virus (ToLCNDV), including potatoes. It is widely distributed in tropical and subtropical regions and transmitted by sap-sucking whiteflies (*Bemisia tabaci* Genn.). This virus is responsible for apical leaf curl disease in potato. The general symptoms of this disease are yellowing, curling, and twisting of leaves, stunting of plants, decrease in tuber production, and reduction of the quality of potato, leading to significant economic losses for farmers [7].

Potato is a versatile and highly nutritious vegetable starchy crop consumed worldwide. One of the most significant nutritional benefits of the potato is its high carbohydrate content, which is crucial for providing energy to the body [8]. However, the emerging challenges of abiotic and biotic stress have significantly affected its quality and nutrition. Moreover, the change in the physiological and biochemical response in plants is associated with the viral infection [9]. However, there are meagre reports on the effect of ToLCNDV in physiological and biochemical response in potato plants. Therefore, to understand the morphological, physiological, and biochemical response of virus-induced mechanisms in potato plants, various aspects of plant growth and development are necessary. Earlier reports on other viruses suggest that potato plant may exhibit changes in the plant architecture with distorted leaves and stunted growth [10]. The viral infection affects the potato plant’s carbohydrate metabolism by altering the synthesis of starch in the leaves and its deposition in source/ tuber tissues. The change in the plant morphological traits might be due to the alteration in the phytohormonal imbalance, which is probably affected by the virus infestation [9].

Earlier reports suggest that the alteration of plant metabolism by viral infection leads to a reduction of photosynthetic activities, ultimately lowering yield. The virus also affects the source–sink relationship by affecting nutrient uptake and transport, leading to nutrient deficiencies in the plant [11]. The biochemical responses of the virus-infected plant were reported to have altered the metabolism that modulates the metabolite levels such as sugar, amino acid, protein, sucrose, starch, pigments, and other molecules [12]. The viral infection in potato plants leads to the synthesis of reactive oxygen species (ROS), which can cause oxidative damage in the plant tissue and affect the plant immune system [13]. In response to a viral infection, plants synthesize small interfering RNAs (siRNAs), which activate systemic acquired resistance (SAR). Therefore, understanding the morpho-physiological and biochemical responses of virus-infected potato plants is essential for developing cultivars resistant to ToLCNDV [4,14,15]. Similarly, the infection due to the virus leads to the synthesis of reactive species molecules, and the resistant/tolerance level of the plant is decisive in detoxifying the molecules. Virus infection can induce the production of reactive oxygen species (ROS), which can damage proteins, membranes, and nucleic acids. This can lead to impaired plant growth and development. However, plants have developed antioxidant systems to counteract ROS, such as superoxide dismutase (SOD), catalase, and peroxidase [3].

The virus infection can alter gene expression and signal transduction, which might be via the mitogen-activated kinase cascade and phytohormonal pathway. Virus infection in potato can change the activity and expression of antioxidant enzymes, which can affect the plant’s ability to cope with oxidative stress [16,17]. Additionally, virus infection can affect the expression of genes involved in ROS scavenging and the biosynthesis of antioxidants, such as ascorbic acid, glutathione, and phenolic compounds.

These changes can alter the plant’s redox balance, further exacerbating oxidative stress [17]. The aforementioned changes impact regulating oxidative stress by altering synthesis and scavenging ROS, ultimately affecting the genes responsible for the biosynthesis of antioxidants and their enzymes. The report suggested that potato virus Y infection in potato plants was shown to induce oxidative stress that ultimately affects the growth and yield of the plant [16]. On the contrary, the potato virus X (PVX) infection tends to improve plant defence response toward environmental stresses [18]. Previous reports suggested that superoxide restriction in PVX-infected leaves by supplementation of antioxidants (superoxide dismutase and catalase) partially suppresses resistance along with the appearance of necrotic symptoms resembling a hypersensitive response [19]. In another study, PVY inoculation in potato variety Red Scarlet increased the catalase activity. Likewise, the maximum activity of catalase was observed after co-inoculation with PVS and PVM [20]. Overall, understanding the stress-responsive role of viruses in potato requires studying the molecular and cellular changes that occur in infected plants, including the expression of stress-related genes and proteins and the activity of enzymes and signalling pathways involved in stress responses. This knowledge can help develop strategies for controlling virus infections and improving plant resilience under stress conditions. In view of the above-discussed points, research work was undertaken with the following objectives to gain a comprehensive understanding of the morpho-physiological and biochemical responses elicited by virus-induced mechanisms in potato plants: to investigate the alterations in carbohydrate metabolism pathways and unravel the mechanisms by which virus infection affects these processes in potato plants. The novelty of this study lies in its comprehensive examination of the effects of ToLCNDV-potato infection on various aspects of potato plants, such as morphological parameters, photosynthetic pigment system, oxidative metabolite leakage, starch metabolism, and antioxidant activity. The study sheds light on potential mechanisms underlying ToLCNDV-potato resistance and provides valuable information for future breeding efforts to develop tolerant potato varieties by comparing the responses of susceptible and resistant cultivars.

## 2. Materials and Methods

### 2.1. Pure Virus Culture and Planting Material

Potato cultivars, Kufri Pukhraj (yellow, ovoid tubers with shallow-medium eyes and yellow flesh, susceptible to ToLCNDV-potato) and Kufri Bahar (white-cream, ovoid tubers with medium eyes, white flesh, tolerant to ToLCNDV-potato) were used in this study. These cultivars were maintained in tissue culture at the Division of Plant Protection-CPRI Shimla. The resistance and susceptibility of these cultivars to the virus were previously established through transcriptome studies. The virus (isolate JAL-10, DNA A) was obtained in pure culture from an insect-proof virus culture facility at ICAR-CPRI Shimla, where it is maintained on potato plants (KC874509.1). To initiate the experiment, sprouting was induced in healthy tubers of cultivars Kufri Pukhraj and Kufri Bahar. Agro-inoculation was used through tuber sprout methods followed by stem injection. Fifteen tubers of each cultivar were planted under controlled conditions (temperature 18–22 °C, relative humidity 80–85%) to analyse potato morphology and quality parameters.

### 2.2. Virus Inoculation Studies in Potato

In a previous experiment at the Molecular Laboratory, ICAR-CPRI Shimla, infectious clones of ToLCNDV were constructed and efficacy was studied on potato and *Nicotiana benthamiana* [7]. The studies involved the germination of seed tubers, which were then inoculated with a mixture of *Agrobacterium* cells containing 1:1 ratio of DNA A and DNA B. This artificial inoculation process took place in a rotary shaker for a duration of 4 h. After pricking the sprouts, the inoculated tubers were sown in a sterile pot mixture. Fifteen days after sowing, the grown plants were subjected to a second round of inoculation. Agrobacterium cells were injected into the stems of the plants. The entire process was carried out using four replicates for each treatment, and the experiment was repeated three times to ensure reliability and consistency of results. Sampling was performed after 20 days of inoculation. Throughout the experiment, both the inoculated plants and control plants were maintained under controlled conditions. The virus-inoculated plants were monitored for symptom development. The control (non-inoculated) plants were also maintained alongside infected plants. Four weeks after transplanting, leaves of Kufri Pukhraj and Kufri Bahar were collected and tested for the presence of the virus. According to the manufacturer’s instructions, total DNA was extracted from the potato leaves using the GenEluteTM Plant Genomic DNA kit (Sigma-Aldrich, St. Louis, MO, USA). The quality and concentration of the extracted DNA were measured using a Nanodrop 2000 spectrophotometer (Thermoscientific, Leon-Rot, Germany). The virus was detected using coat-protein-specific primer pairs (LCVCPF1/LCVCPR1) and PCR conditions similar to those previously described [7].

### 2.3. Estimation of Photosynthetic Pigments

The chlorophyll and carotenoid contents of the uppermost fully expanded leaves were determined using the method of Hiscox and Israelstan (1979) [21] and expressed as mg g^−1^ of leaf fresh weight using a UV-Visible spectrometer (model UV-160A, Shimadzu, Kyoto, Japan). In addition, three plants from each pot were assessed for growth characteristics such as plant height, dry matter of tubers, and stem diameter.

### 2.4. Estimation of Proline, MDA, LWC, and REC

Proline was measured according to the method described by [20]. Method was used with a few modifications [22]. One gram of potato leaf material was ground in a mortar, followed by 2 mL of chilled trichloroacetic acid (TCA) solution and quartz sand. A 10 min centrifuge at 4000 rpm was performed on the homogenate after the sample was further ground. A standard for OD determination was prepared by adding 2 mL of distilled water to another test tube with 2 mL of the supernatant transferred to it. After adding 2 mL of 0.6% thiobarbituric acid (TBA) solution to both tubes, they were placed in boiling water for 15 min. After cooling, the samples were centrifuged again, and OD values were measured at 532 nm, 600 nm, and 450 nm using the supernatant. We calculated the MDA concentration by using the formula: MDA concentration (μmol L^−1^) = 6.45 × (OD532 − OD600) − 0.56 × OD450. To calculate RWC (%) for potato leaves, multiply (FW − DW) by (FW − DW)/FW, where FW represents the fresh weight and DW represents the dry weight. To determine their relative electrical conductivity, leaf wafers from 30 plants were filled with 10 mL of distilled water. Plastic caps were placed on the tubes and water was added. A vacuum was applied to the tubes for 10 min, followed by 1 h at room temperature before measuring the electrical conductivity (S1). A second measurement of electrical conductance (S2) was conducted after the tubes were placed in boiling water for 20 min, cooled, and then placed in ice water for 20 min. Distilled water was also measured for its electrical conductance (S3). Leaf relative electric conductivity (%) was calculated from (S1 − S3)/(S2 − S3) × 100.

### 2.5. Determination of Carbohydrates in Potato Leaves

For determination of soluble sugars, about 50 mg of leaf tissue was ground with liquid nitrogen to form a fine powder. In order to extract the soluble sugars, 1 mL of 80% ethanol was heated at 80 °C for 30 min and then extracted three times. The samples were then cooled and centrifuged for 10 min at 4 °C with 16,000× *g*. Following centrifugation, the supernatants were combined and evaporated. To determine the soluble sugar concentration, the dried residues were resuspended in 0.5 mL of distilled water. In the presence of glucose-6-phosphate dehydrogenase, hexokinase, phosphoglucose isomerase, and invertase were used to determine glucose, fructose, and sucrose contents. Using the Megazyme total starch assay procedure, starch was quantified. DMSO was added to the dried pellet and the starch was resuspended in DMSO and boiled to facilitate gelatinization. The enzymatic hydrolysis was performed using α-amylase and glucoamylase. Each sample was tested in triplicate. In order to extract sugar from leaf tissue, 80% ethanol was used as the extraction solvent. Glucose, fructose, and sucrose analyses were performed on the samples enzymatically. Megazyme total starch assay procedure was used to quantify starch using α-amylase and glucoamylase. Tests were conducted on triplicate samples according to the protocol.

### 2.6. Estimation of Starch Hydrolyzing Enzymes

To assess β-amylase (EC 3.2.1.2) activity, control and infected leaves (ToLCNDV induced infection) were ground into a fine powder using a mortar and pestle. Using a solution containing 50 mM Tris-HCl (pH 8.0), 1 mM Na_2_EDTA, 2% (*m*/*v*) polyvinylpyrrolidone (PVP), and 100 mM cysteine hydrochloride, crude extracts were prepared. After centrifuging at 16,000× *g* and 4 °C for 15 min, the mixture was cooled. A p-nitrophenyl maltotrioside (PNPG3) enzyme activity assay was performed with the resulting supernatant diluted 10-fold with 100 mM MES (pH 6.2) containing 1 mM Na_2_EDTA. The assay was performed according to the manufacturer’s instructions using the betamyl-3 reagent (Megazyme, Bray, Ireland). During incubation, samples were measured at 400 nm for 2 and 4 h at 40 °C to see if p-nitrophenol was produced. The experiment was performed three times.

With a mortar and pestle, we ground 500 mg of control and infected leaves (ToLCNDV induced infection) into a fine powder in liquid nitrogen to test their activity. Crude extract was prepared with a buffer containing 50 mM sodium malate, 50 mM sodium chloride, 2 mM calcium chloride, 2% PVP, and 0.0005% sodium azide (*m*/*v*). For 15 min at 4 °C and 16,000× *g*, the extract was centrifuged. Based on the manufacturer’s instructions, we measured the amylase activities using a PNPG7 substrate (Ceralpha, Megazyme, Wicklow, Ireland). Spectrophotometric measurements were taken at 400 nm after 20 min at 40 °C.

A 500 mg leaf powder extract was extracted in 1 cm^3^ of 50 mM HEPES (pH 7.0), 1 mM Na2EDTA, 4 mM 2-mercaptoethanol, and 0.1 mM phenylmethanesulfonyl fluoride (PMSF). The phosphorylase activity was measured according to [23]. The activity was measured by monitoring the absorbance at 340 nm at 5 min intervals at 30 °C for 30 min and expressed as the production of NADPH.

### 2.7. Estimation of Antioxidant Enzymes

The activity of SOD was measured using spectrophotometry, following the Spychalla and Desborough (1990) [24] method. The CAT activity was determined according to the Lin and Wang (2002) [13] method by monitoring the consumption of H_2_O_2_ (extinction coefficient 39.4 mmol L^−1^ cm^−1^) at 240 nm for 2 min. Finally, the POD activity was determined using the Huseynova et al. (2005) [3] method.

### 2.8. Statistical Analysis

The data were analysed using IBM Corporation’s SPSS version 22.0 (Armonk, NY, USA). Analysis of variance (ANOVA) was performed, and the LSD test (least significant difference) was used to compare the means between treatments. Data obtained from the measurements were analysed using Prism 6.01 software developed by GraphPad Software Inc. in San Diego, CA, USA. The software is a widely used statistical analysis tool that provides a wide range of tests and tools to analyse and interpret data. To determine the impact of two independent variables on the measured parameters, we performed a two-way analysis of variance (ANOVA).

## 3. Results

### 3.1. Symptom Expressions and Molecular Detection of the Virus

**Figure 1:**
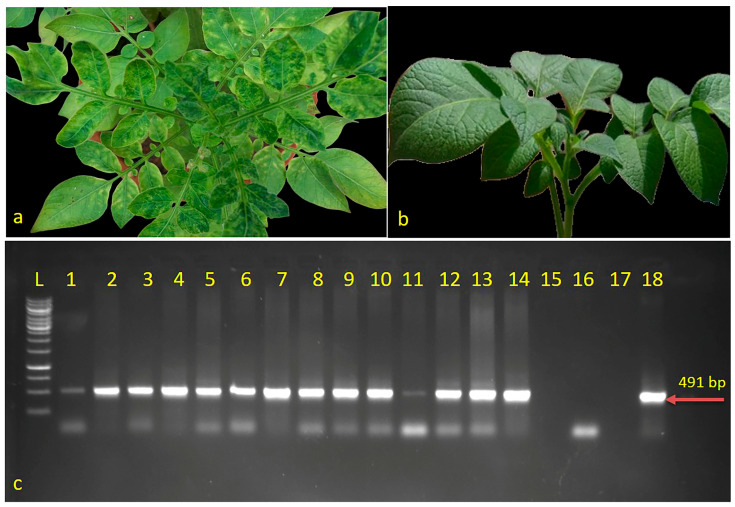
Symptomatic plants of (**a**) Kufri Pukhraj; (**b**) Kufri Bahar; (**c**) RT-PCR mediated detection of infected samples (Kufri Pukhraj); L-1kb Ladder; 1–14 positive samples; 15 and 17 water control; 16 healthy control; 18 known positive.

PALCD (potato apical leaf curl disease) is a virus-borne disease caused by ToLCNDV (tomato leaf curl New Delhi virus), which causes mild chlorosis in potato leaves. As in the previous report by Jeevalatha et al., 2017 [7], out of the cultivar screened for PALCD, Kufri Bahar was highly resistant and Kufri Pukhraj was susceptible. The disease exhibits distinct symptoms, including conspicuous mosaic patterns on the leaves, curling of leaves and upward crinkling, and stunted plant growth. Kufri Pukhraj plants exhibited typical leaf curling and chlorosis symptoms after 15 days of transplantation, although mild chlorosis was evident even after 10 days. In contrast, resistant potato cultivars like Kufri Bahar and control plants of both cultivars showed mild chlorotic symptoms or no symptoms, showing their resistance to the disease (Figure 1a,b).

In this study, all the inoculated plants, including the susceptible Kufri Pukhraj, were found to be infected with ToLCNDV-potato. Both ELISA testing and coat protein-based PCR testing were used for confirmation. In all virus-inoculated plants, PCR amplification using primer sets LCVCPF1 and LCVCPR1 produced a 491 bp band, whereas in the resistant and control plants, there was no amplification (Figure 1c). This finding confirms the presence of the virus in susceptible plants and its absence in the resistant and control plants. This study’s results provide compelling evidence for the presence of ToLCNDV-potato and its association with potato apical leaf curl disease. Moreover, this study highlights the usefulness of ELISA and PCR-based detection methods in confirming the presence of viral pathogens in plants. Using these methods, researchers can quickly and accurately diagnose plant viral infections, which is crucial for controlling the spread of plant diseases and ensuring food security [5]. It has been demonstrated in our study that ToLCNDV-potato causes potato apical leaf curl disease. This study emphasizes the importance of accurate and quick methods of diagnosing plant viral infections such as ELISA and PCR-based detection in the field of plant pathology. In the future, this research could be used to help plant pathologists and farmers take the necessary measures to prevent the spread of plant diseases and ensure the health of crops.

### 3.2. Modification of Potato Morphological Parameters by PALCV

It has been suggested that viruses can negatively affect the plant’s morphological traits [2,24]. In our study, we found PALCV infection in both Kufri Pukhraj and Kufri Bahar reduces plant height by 13.4% and 10.8%, respectively (Table 1, Tables S1 and S2). It is possible for the virus to disrupt physiological functions, such as hormone production and growth regulation, within plants. The reduction of cell division and elongation might be responsible for stunted growth and shorter plant height. There are various symptoms that may indicate an infection caused by ToLCNDV, such as stunting, curling, yellowing, and wilting of leaves as a result of the presence of the virus. Due to these symptoms, it is probable for the plant to develop and maintain an unhealthy root system, which will hamper its growth and height [25].

The morphology of potato plants infected with ToLCNDV-potato was negatively affected. The leaves, particularly those located at the apex, displayed symptoms related to mottling, chlorosis, and curling. In our experiment, we harvested the tubers from two potato cultivars, Kufri Pukhraj and Kufri Bahar, and analysed their dry matter content over time in order to see how ToLCNDV-potato infection affected their dry matter content. It was found that root infection with ToLCNDV-potato caused a significant decrease in dry matter content in both cultivars (Tables S1 and S3, *p* < 0.05) as a result of the infection with ToLCNDV-potato. A decrease of 21.66% in the dry matter content was observed in Kufri Pukhraj, while a reduction of 18.59% was observed in Kufri Bahar (Table 1).

When it comes to potato plants, the diameter of the stem plays a significant part in determining the general health and vigour of the plant. A stronger, more robust plant that can handle environmental stressors such as wind, disease, and pests will have a thicker stem diameter [4]. In our study, the stem diameter in Kufri Pukhraj was reduced by 17.26% and in Kufri Bahar, it was reduced by 8.61% (Table 1, Tables S1 and S4). As Kufri Bahar was a resistant cultivar therefore the reduction was less as compared to the Kufri Pukhraj cultivar. The study found that the resistant potato cultivar, Kufri Bahar, showed less severe reduction in plant height, stem diameter, and dry matter content of potato compared to the susceptible cultivar, Kufri Pukhraj, when infected with ToLCNDV.

### 3.3. Effect of ToLCNDV-Potato on the Photosynthetic Parameters of Potato Plant

**Figure 2:**
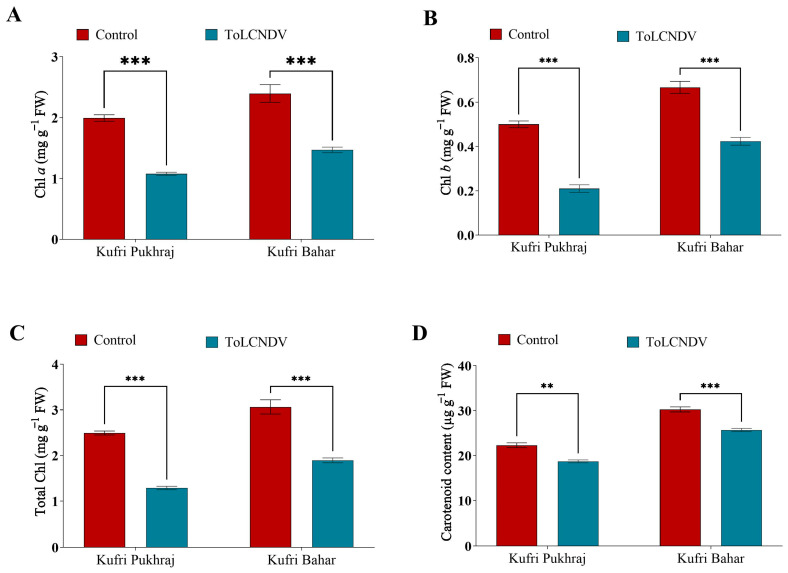
Effect of potato apical leaf curl disease on (**A**) Chl *a*, (**B**) Chl *b*, (**C**) total Chl and (**D**) carotenoid content in potato leaves of Kufri Pukhraj and Kufri Bahar. Bars represent mean values for parameters. The statistical significance of the difference between the means across varieties. All data are represented as mean values with standard error of three replications. Tukey’s multiple comparison test showed that mean values of ToLCNDV-potato infection were significantly different (*p* < 0.001) from control (healthy) potato tubers of Kufri Pukhraj and Kufri Bahar. ** and *** mean values are significantly different (*p* < 0.01 and *p* < 0.001 respectively).

In response to ToLCNDV-potato a significant reduction (*p* < 0.05) of chlorophyll (Chl) *a* content by 46% was observed in Kufri Pukhraj and about 39% in Kufri Bahar (Figure 2A and Table S1). The results indicate that the biotic stress caused by ToLCNDV infection in potato plants resulted in a significant reduction (Table S5) of chlorophyll a content in the leaves. This reduction can be attributed to the destruction of chloroplasts caused by the viral infection. The Chl *a* content of Kufri Pukhraj was 1.9 mg g^−1^ FW which was reduced to 1.08 mg g^−1^ FW after PALCV infection. A similar result was obtained when TolCNDV infected Kufri Bahar where the Chl *a* content was reduced from 2.4 mg g^−1^ FW to 1.47 mg g^−1^ FW (Figure 2A and Tables S1 and S5). To our knowledge, this is the first report regarding the effect of ToLCNDV infection on Chl *a* content in potato leaves. The Chl *b* content of Kufri Pukhraj was 0.50 mg g^−1^ FW, which was reduced to 0.21 mg g^−1^ FW after ToLCNDV infection in potato. Similarly, when TolCNDV infected Kufri Bahar the Chl *b* content was reduced from 0.67 mg g^−1^ FW to 0.42 mg g^−1^ FW (Figure 2B). In response to ToLCNDV-potato a significant reduction (*p* < 0.05; Table S1, and S6) of Chl *b* content by 58% was observed in Kufri Pukhraj and about 37% in Kufri Bahar (Figure 2B). When the total Chl was estimated the trend was similar to Chl *a* and Chl *b*. Our result revealed that the total Chl content was reduced by 48% and 38% in Kufri Pukhraj and Kufri Bahar, respectively (Figure 2C and Tables S1 and S7). The higher reduction of total Chl was observed in Kufri Pukhraj, which was susceptible and lower reduction was observed in Kufri Bahr, which was a tolerant cultivar. The tolerant cultivar showed traits of maintenance of Chl in the leaves by enhancing the regulatory mechanism of slow degradation of Chl under viral infection. In contrast, a significant decrease of approximately 16% and 15% in total carotenoid content was observed in Kufri Pukhraj and Kufri Bahar, respectively (Figure 2D and Table S8). The initial carotenoid content in the control leaves of Kufri Pukhraj and Kufri Bahar was 22.29 and 30.26 µg g^−1^ FW, which was reduced to 18.70 and 25.68 µg g^−1^ FW, respectively.

### 3.4. Effect of ToLCNDV-Potato on Proline, MDA and Relative Electrical Conductivity of Potato Plant

**Figure 3:**
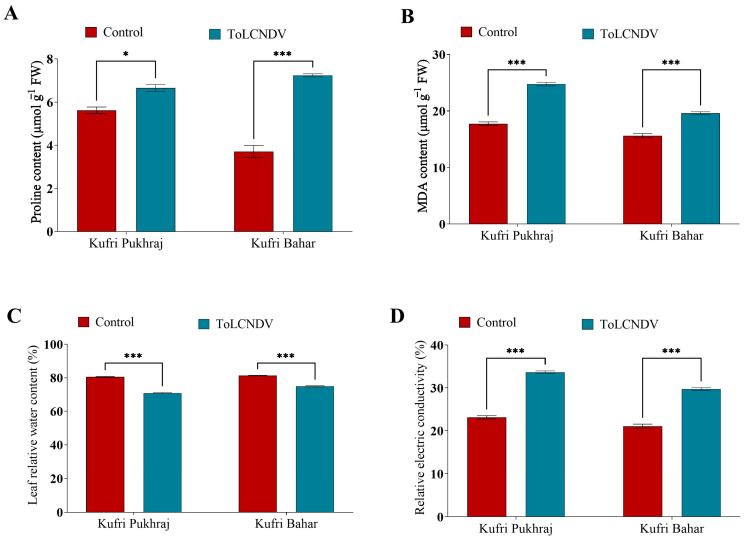
Effect of potato apical leaf curl disease on (**A**) proline content, (**B**) malondialdehyde (MDA) content, (**C**) relative electrical conductivity, and (**D**) relative electric conductivity in potato leaves of Kufri Pukhraj and Kufri Bahar. Bars represent mean values for parameters. The statistical significance of the difference between the means across varieties. All data are represented as mean values with standard error of three replications. Tukey’s multiple comparison test showed that mean values of ToLCNDV-potato infection were significantly different (*p* < 0.001) from control (healthy) potato tubers of Kufri Pukhraj and Kufri Bahar. * and *** mean values are significantly different (*p* < 0.05 and *p* < 0.001 respectively).

The infection due to ToLCNDV-potato leads to enhancement of proline content in both the cultivars, viz., Kufri Pukhraj and Kufri Bahar. There was a significant increase (*p* < 0.05; Table S1) in the proline content from 5.62 to 6.65 µmol g^−1^ FW in Kufri Pukhraj, which was about 18% increase as compared to the control (Figure 3A and Tables S1 and S9). Similarly, the increase in the proline content due to PALCV infection in Kufri Bahar was about 95% as compared to control plants (Figure 3A and Table S9).

In our study, the MDA content was reported to be enhanced by 40% and 25% in Kufri Pukhraj and Kufri Bahar, respectively. The higher MDA content in Kufri Pukhraj (24.74 μmol g^−1^ FW) was reported under TolCNDV infection, while in Kufri Bahar the lower MDA content of 19.60 μmol g^−1^ FW was reported (Figure 3B and Tables S1 and S10).

In response to ToLCNDV-potato a significant reduction (*p* < 0.05) of leaf relative water content by 12% was observed in Kufri Pukhraj and about 8% in Kufri Bahar (Figure 3C; Tables S1 and S11). The leaf relative water content of Kufri Pukhraj was lowered from 80.59% to 70.90% due to ToLCNDV and in Kufri Bahar, the leaf relative water content was lowered from 81.40% to 74.92%. Our result showed that the viral infection due to ToLCNDV leads to the lowering of leaf relative water content in both the cultivars.

The relative electrical conductivity of both cultivars under viral infection was suggested to be enhanced. In Kufri Pukhraj, the increment in the relative electrical conductivity was reported to be 45% higher, while in Kufri Bahar the increase in relative electrical conductivity was reported to be 41% higher as compared to their respective control (Figure 3D and Tables S1 and S12). The relative electrical conductivity of Kufri Pukhraj was enhanced from 23.13% to 33.67% due to PALCV infection. On the contrary, the infection due to PALCV in Kufri Bahar leads to the enhancement of relative electrical conductivity from 21.05% to 29.71% (Figure 3D and Table S12).

### 3.5. Effect of ToLCNDV-Potato on Carbohydrate Profile of Potato Plant

**Figure 4:**
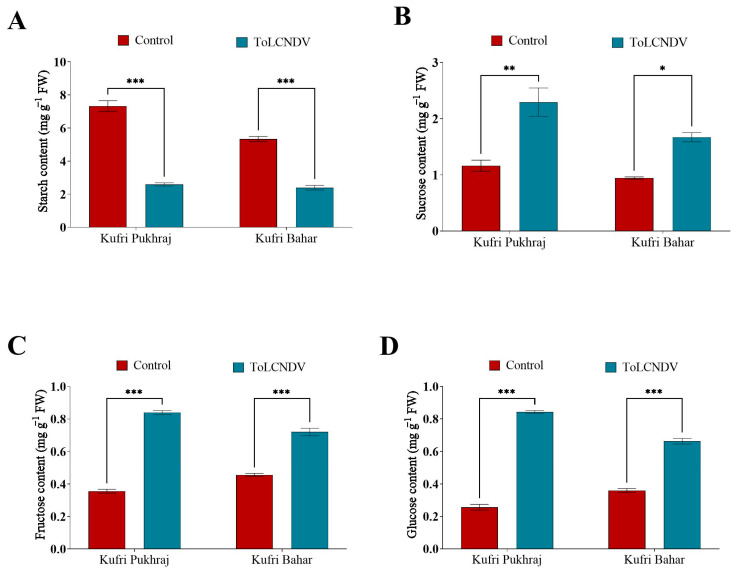
Effect of potato apical leaf curl disease on (**A**) starch content, (**B**) sucrose content, (**C**) fructose, and (**D**) glucose content in potato leaves of Kufri Pukhraj and Kufri Bahar. Bars represent mean values for parameters. The statistical significance of the difference between the means across varieties. All data are represented as mean values with standard error of three replications. Tukey’s multiple comparison test showed that mean values of ToLCNDV-potato infection were significantly different (*p* < 0.001) from control (healthy) potato tubers of Kufri Pukhraj and Kufri Bahar. *, ** and *** mean values are significantly different (*p* < 0.05, *p* < 0.01, and *p* < 0.001 respectively).

Carbohydrate metabolism plays an important role in the plant defence mechanism under the biotic stress condition. Our results suggest that the infection due to ToLCNDV was shown to have a reduction (Tables S1 and S13) in starch content in leaves by 64% and 55% in Kufri Pukhraj and Kufri Bahar, respectively (Figure 4A). The reduction in starch content was observed as early as 45 days after infection and became more pronounced as the infection progressed. On the contrary, the experimental output suggests that the infection due to ToLCNDV significantly enhanced (Tables S1 and S14) non-reducing sugars such as sucrose (Figure 4B). The sucrose contents in the leaves of Kufri Pukhraj and Kufri Bahar were reported to be 1.16 and 0.94 mg g^−1^ FW, which was enhanced to 2.29 and 1.67 mg g^−1^ FW after ToLCNDV infection in both the cultivars.

Similarly, glucose and fructose contents were also reported to be enhanced due to ToLCNDV infection in potato leaves of Kufri Pukhraj and Kufri Bahar (Figure 4A,B). The enhancement of glucose was reported to be 2.28 and 0.8 fold in the leaves of Kufri Pukhraj and Kufri Bahar, respectively (Tables S1, S15, and S16). In Figure 4C, the reducing sugar content of two potato cultivars (Kufri Pukhraj and Kufri Bahar) was measured for both control and ToLCNDV-potato treatments. The results showed a significant (*p* < 0.01) increase in reducing sugar content (glucose) in both cultivars infected with ToLCNDV-potato. Interestingly, a higher fructose content was reported in the leaves of Kufri Pukhraj (1.3-fold), and lower fructose content was suggested in Kufri Bahar (0.6 fold) (Figure 4D).

The total soluble sugar content signifies a higher accumulation of soluble sugar in the cytoplasm due to abiotic and biotic stress. In our study, the total soluble sugar content was reported to be enhanced by 18% and 13% in Kufri Pukhraj and Kufri Bahar. The higher total soluble sugar content in Kufri Pukhraj (71.26 mg g^−1^ DW) was reported under ToLCNDV infection, while in Kufri Bahar a lower total soluble sugar content of 60.90 mg g^−1^ DW was reported. The aforementioned results indicate that ToLCNDV infection affects carbohydrate metabolism in potato leaves.

### 3.6. Effect of ToLCNDV-Potato on Carbohydrate Metabolic Enzymes of Potato Plant

**Figure 5:**
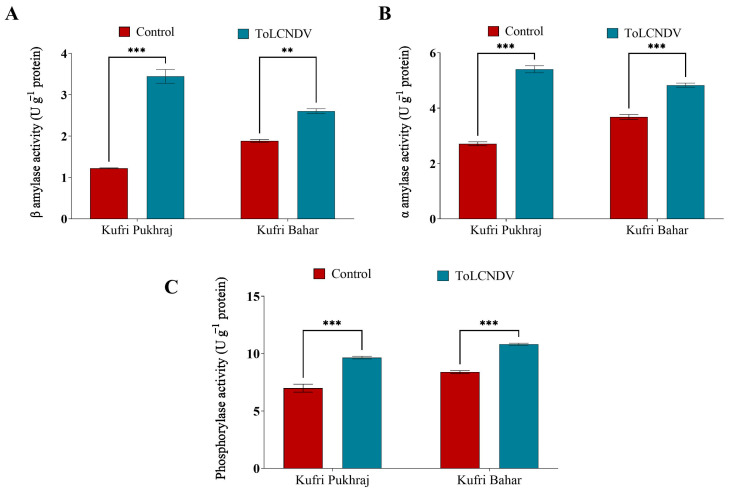
Effect of potato apical leaf curl disease on (**A**) β-amylase, (**B**) α-amylase, and (**C**) phosphorylase activities in potato leaves of Kufri Pukhraj and Kufri Bahar. Bars represent mean values for parameters. The statistical significance of the difference between the means across varieties. All data are represented as mean values with standard error of three replications. Tukey’s multiple comparison test showed that mean values of ToLCNDV-potato infection were significantly different (*p* < 0.001) from control (healthy) potato tubers of Kufri Pukhraj and Kufri Bahar. ** and *** mean values are significantly different (*p* < 0.01, and *p* < 0.001 respectively).

In potato leaves, β-amylase is involved in the breakdown of starch to provide energy for growth and other metabolic processes [26]. Our result suggested that the β-amylase activity of Kufri Pukhraj and Kufri Bahar was reported to be 1.22 and 1.88 U g^−1^ protein which was enhanced to 3.44 and 2.60 U g^−1^ protein due to ToLCNDV infection, respectively (Figure 5A and Tables S1 and S17).

Our results suggest that α-amylase activity was enhanced by 1.0 fold and 0.30 fold in Kufri Pukhraj and Kufri Bahar, respectively (Figure 5B and Tables S1 and S18). The maximum increase in the α-amylase activity was reported to be higher in the Kufri Bahar, compared to Kufri Pukhraj. As Kufri Pukhraj is a more susceptible cultivar, the infection due to ToLCNDV might lead to higher α-amylase activity.

Phosphorylase is a key enzyme in carbohydrate metabolism in potato leaves, as it is involved in both the breakdown and synthesis of carbohydrates, which are critical sources of energy for the plant [27]. Our result suggested the phosphorylase activity of Kufri Pukhraj and Kufri Bahar was reported to be 6.99 and 8.39 U g^−1^ protein which was enhanced to 9.85 and 10.81 U g^−1^ protein due to ToLCNDV infection, respectively (Figure 5C and Tables S1 and S19). Phosphorylase is responsible for the transfer of glucosyl units from glucose-1-phosphate to the non-reducing end of α-1,4-D-glucan chains.

### 3.7. Effect of ToLCNDV-Potato on Antioxidant Enzyme Activity of Potato Plant

**Figure 6:**
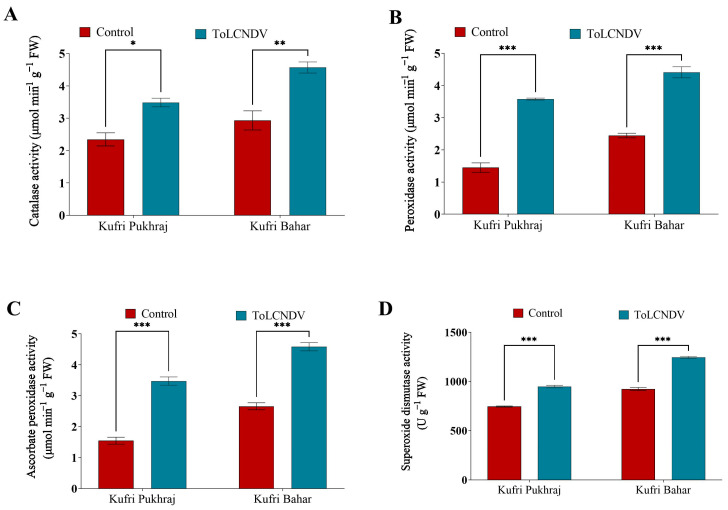
Effect of potato apical leaf curl disease on (**A**) catalase activity, (**B**) peroxidase activity, (**C**) ascorbate peroxidase activity, and (**D**) superoxide dismutase in potato leaves of Kufri Pukhraj and Kufri Bahar. Bars represent mean values for parameters. The statistical significance of the difference between the means across varieties. All data are represented as mean values with standard error of three replications. Tukey’s multiple comparison test showed that mean values of ToLCNDV-potato infection were significantly different (*p* < 0.001) from control (healthy) potato tubers of Kufri Pukhraj and Kufri Bahar. *, ** and *** mean values are significantly different (*p* < 0.05, *p* < 0.01, and *p* < 0.001 respectively).

The infection caused by ToLCNDV-potato results in a notable increase in catalase activity in both cultivars, namely Kufri Pukhraj and Kufri Bahar, by approximately 48% and 55%, respectively. This increase in catalase activity is significant (Tables S1 and S20), as it elevates the activity from 2.35 to 3.49 mmol min^−1^ g^−1^ FW in Kufri Pukhraj and from 2.93 to 4.57 mmol min^−1^ g^−1^ FW in Kufri Bahar, when compared to the control (Figure 6A).

The ToLCNDV infection in both the cultivars viz., Kufri Pukhraj and Kufri Bahar suggested significant enhancement of the peroxidase activity. The increases in the peroxidase activity in Kufri Pukhraj and Kufri Bahar were reported to be 146% and 80%, respectively (Tables S1 and S21). The value of peroxidase activity reported in Kufri Pukhraj was 1.45 mmol min^−1^ g^−1^ FW and that was enhanced to 3.58 mmol min^−1^ g^−1^ FW (Figure 6B). Likewise, under ToLCNDV infection, the peroxidase activity was found to increase from 2.45 to 4.41 mmol min^−1^ g^−1^ FW under ToLCNDV infection.

The two cultivars, Kufri Pukhraj and Kufri Bahar, both showed a significant increase (Tables S1 and S22) in ascorbate peroxidase activity when infected with PALCV. In Kufri Pukhraj, the enzyme activity increased from 1.54 to 3.47 mmol min^−1^ g^−1^ FW, representing a 125% increase compared to the control. Meanwhile, Kufri Bahar showed an enhancement of ascorbate peroxidase activity from 2.66 to 4.88 mmol min^−1^ g^−1^ FW, representing a 72% increase in the enzyme’s activity (Figure 6C). This suggests that PALCV infection has a positive effect on ascorbate peroxidase activity in both cultivars, with Kufri Pukhraj demonstrating a greater increase in activity compared to Kufri Bahar.

The infection caused by ToLCNDV-potato was found to increase the activity of the antioxidant enzyme superoxide dismutase (SOD) in two potato cultivars (Tables S1 and S23), Kufri Pukhraj and Kufri Bahar. In Kufri Pukhraj; SOD activity was increased by approximately 27% in Kufri Pukhraj and in Kufri Bahar and it was increased by about 34.7%, when compared to the control. The increase in SOD activity in response to ToLCNDV-potato infection is significant, as it elevates the SOD activity levels from 745.40 to 948.15 U g^−1^ FW in Kufri Pukhraj and from 923.70 to 1245.03 U g^−1^ FW in Kufri Bahar (Figure 6D).

## 4. Discussion

PALCD (potato apical leaf curl disease) is a virus-borne disease caused by ToLCNDV (tomato leaf curl New Delhi virus), which causes mild chlorosis in potato leaves. As in the previous report by Jeevalatha et al., 2017, out of the cultivar screened for PALCD, Kufri Bahar was highly resistant and Kufri Pukhraj was susceptible [7]. The disease exhibits distinct symptoms, including conspicuous mosaic patterns on the leaves, curling of leaves and upward crinkling, and stunted plant growth. It has been suggested that viruses can negatively affect the plant’s morphological traits [2,24]. In our study, we found PALCV infection in both Kufri Pukhraj and Kufri Bahar reduced plant height by 13.4% and 10.8%, respectively (Table 1, Tables S1 and S2). It is possible for the virus to disrupt physiological functions, such as hormone production and growth regulation, within plants. The reduction of cell division and elongation might be responsible for stunted growth and shorter plant height. The presence of ToLCNDV infection can lead to several observable symptoms in plants, including stunted growth, leaf curling, yellowing, and wilting. These symptoms can adversely affect the plant’s root system, hindering its overall health and impeding its growth and height [25].

A previous study on sweet potato also showed similar findings, where the infection caused by sweet potato chlorotic stunt virus (*SPCSV*) and sweet potato feathery mottle virus (*SPFMV*) resulted in a reduction in tuber fresh weight [28]. The reduction in dry matter content observed in this study can be attributed to the impact of ToLCNDV-potato infection on the plant’s ability to photosynthesize and produce energy, as well as the breakdown of cellular components [29]. Additionally, the reduced dry matter content may also be a result of the virus interfering with the plant’s ability to uptake and use water and nutrients. The study found that the resistant potato cultivar, Kufri Bahar, showed less severe reduction in plant height, stem diameter, and dry matter content of potato compared to the susceptible cultivar, Kufri Pukhraj, when infected with ToLCNDV. This indicates that the resistance mechanism in Kufri Bahar may provide some level of protection against the negative impacts of virus-induced reductions in plant health. Although resistance to virus infection may not necessarily lead to an increase in yield, it can still offer some protection against the adverse effects on plant health and productivity [30]. These results suggest that further research could investigate the mechanisms that underlie this protection and explore ways to improve crop resilience by enhancing these mechanisms.

Our result revealed that the total Chl content was reduced by 48 and 38% in Kufri Pukhraj and Kufri Bahar, respectively. The higher reduction of total Chl was observed in Kufri Pukhraj, which was susceptible and lower reduction was observed in Kufri Bahr, which was a tolerant cultivar. The reduction in the carotenoid content might be due to degradation of chloroplast which is caused due to viral infection in the leaves. The infection due to ToLCNDV-potato leads to enhancement of proline content in both the cultivar, viz., Kufri Pukhraj and Kufri Bahar. The higher accumulation of proline might be due to the resistant nature of Kufri Bahar against the PALCV infection. Proline is a molecule that is generally induced in higher amounts due to abiotic and biotic stress. The earlier reports suggest that a higher accumulation was observed under abiotic stress [31]. But the biotic stress also induces proline accumulation, which was confirmed in our experiment.

The MDA content signifies higher lipid peroxidation in plant due to abiotic and biotic stress. Under stress conditions, such as exposure to environmental factors like extreme temperatures or pollutants, plant cells may experience an increase in the production of reactive oxygen species (ROS) molecules. These ROS can cause damage to the membrane lipids of the cell, leading to an enhancement of lipid peroxidation. Lipid peroxidation is a process where the lipids in the cell membrane react with ROS, resulting in the generation of toxic byproducts such as malondialdehyde (MDA) [31]. MDA content can be used as an indicator of the degree of oxidative stress that a plant is experiencing. Therefore, measuring the MDA content in plant tissue can provide insight into the level of damage caused by oxidative stress. This information can be useful in determining the appropriate course of action to mitigate the effects of stress on the plant. Our results showed that the viral infection due to ToLCNDV leads to the lowering of leaf relative water content in both the cultivars. This might be due to the development of flaccid conditions due to viral infection in potato plants, where the plant response mechanism is hijacked by virus which leads to destruction of cell organelle and ultimately leads to lowering of leaf relative water content [32]. Relative electrical conductivity is a commonly used indicator of the degree of cell membrane damage or permeability under stress conditions. When cells are exposed to stress, such as pathogen infection, the cell membrane can become damaged, resulting in the leakage of electrolytes from the cell. This electrolyte leakage can be quantified by measuring the relative electrical conductivity of the cell. In the case of PALCV infection, it has been suggested that the virus enhances the production of reactive oxygen species (ROS) molecules, which can damage the cell membrane content and lead to increased electrolyte leakage from the cells of infected potato plants [33,34]. By measuring the relative electrical conductivity of these cells, researchers can gain insight into the degree of membrane damage caused by PALCV infection.

Carbohydrate metabolism plays an important role in plant defence mechanism under biotic stress condition. Our results suggest that the infection due to ToLCNDV was shown to give a reduction in starch content in leaves in Kufri Pukhraj and Kufri Bahar, respectively. However, there is limited research on the impact of ToLCNDV infection specifically on sucrose content in potato leaves. The enhancement in sucrose content might be due to the disruption of the phloem transport system caused by the viral infection [35]. Similarly, glucose and fructose contents were also reported to be enhanced due to ToLCNDV infection in potato leaves of Kufri Pukhraj and Kufri Bahar. The higher glucose content in the leaves might be due to the degradation of starch in the leaves that releases free glucose [32,35,36]. According to Herbers et al. (2000), virus infection can lead to an increase in sugar levels in plants, which is often associated with resistance [37]. The total soluble sugar content signifies a higher accumulation of soluble sugar in the cytoplasm due to abiotic and biotic stress. In our study, the total soluble sugar content was reported to be enhanced in Kufri Pukhraj and Kufri Bahar. The aforementioned results indicate that ToLCNDV infection affects carbohydrate metabolism in potato leaves. In addition to reducing starch content, it promotes the accumulation of sucrose, glucose, and total soluble sugars, possibly through downregulation of starch-related genes [37,38]. As a result of the viral infection, the plant’s carbohydrate composition changes, reflecting the plant’s response. This highlights the complex interaction between viruses and plant metabolism [36,38].

The higher activity of β-amylase in both the cultivars due to ToLCNDV infection might be due to the attempt of the plant to resist the viral infection, which leads to starch degradation and the formation of free glucose levels in potato leaves [39]. Further research is needed to fully understand the relationship between viral infection and plant β-amylase activity. Our results also correlate with the higher glucose and fructose content in the virus-infected plant potato leaves, which might be due to higher activity of α-amylase activity in potato leaves [39]. A complex regulation of carbohydrate metabolism in potato leaves is explained by enhanced activities of amylases, and phosphorylases in response to ToLCNDV infection [40]. The results of this study provide an insight into the molecular mechanisms by which plants respond to viral infections and highlight the importance of starch breakdown and glucose availability in defence mechanisms and energy production during stressful circumstances.

Catalase activity is higher in both cultivars, which suggests the plants are actively fighting the oxidative stress caused by the viral infection. In addition to the catalase activity increase, reactive oxygen species (ROS) are neutralised and plant cells are protected [41]. Peroxidases are part of the plant’s defence system against viral infections, and they play a crucial role in potato plants infected with viruses. In potato plants infected with viruses, peroxidases are involved in several defence mechanisms. One of the primary roles of peroxidases during viral infection is the detoxification of reactive oxygen species (ROS) that are produced during the plant’s defence response [42]. ROS can damage plant cells, and peroxidases help to neutralize them, reducing the extent of cell damage. Enhanced catalase, ascorbate peroxidase, peroxidase, and superoxide dismutase activities in response to ToLCNDV infection demonstrate the plants’ ability to counteract oxidative stress. In addition to neutralizing reactive oxygen species, these antioxidant enzymes help protect plant cells against damage [42]. These findings suggest that the plants are mounting a robust antioxidant defence reaction in order to counteract the negative effects of the viral infection.

## 5. Conclusions

**Figure 7:**
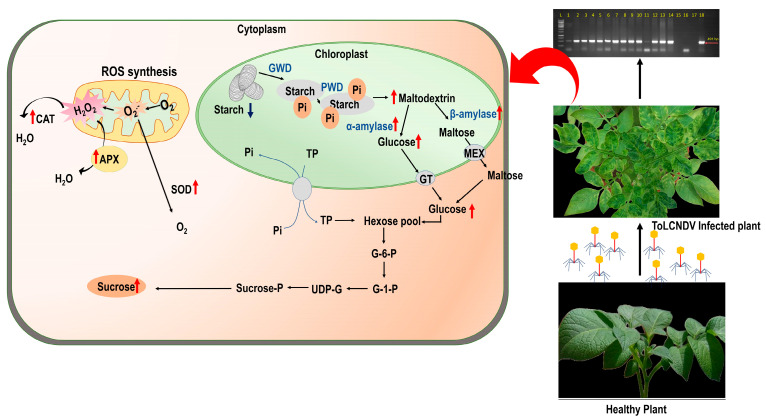
Schematic model of ToLCNDV-potato infection and associated alterations in plant pathophysiological parameters in diseased potato plant.

Based on our study, infection by ToLCNDV-potato virus negatively affected potato plants’ photosynthetic parameters. Both resistant (Kufri Bahar) and susceptible (Kufri Pukhraj) cultivars showed significant reductions in chlorophyll (Chl) a and chlorophyll b content. In Kufri Pukhraj, chlorophyll content decreased by 46%, while in Kufri Bahar, it decreased by 39% and in Kufri Bahar, 58% and 37%, respectively. Plant growth and productivity may be negatively affected by these changes in chlorophyll content, which may indicate a decreased ability to conduct photosynthesis. In addition to this, the infected plants displayed mottling, chlorosis, and curling symptoms, particularly at the apical regions of the leaves. This further indicates that the infection is negatively impacting the health and development of the plants. In order to prevent the negative effects of ToLCNDV-potato virus on potato plants, monitoring and preventing its spread are important (Figure 7). There are significant implications for potato production and breeding because of these findings. As a result of ToLCNDV-potato infection, plants can have reduced productivity and yields due to the reduced photosynthetic capacity. Also, infected plants may have poorer tuber quality, affecting the market value and usability of the tubers. It is crucial to implement monitoring and prevention strategies to limit the spread of ToLCNDV-potato so that negative effects on potato production can be mitigated. Early detection and prompt action can help minimize its impact on plant health and overall crop productivity. Furthermore, future research should focus on elucidating the mechanisms responsible for the observed adverse effects and developing mitigation strategies. By understanding the molecular and physiological pathways affected by the virus, we will be able to develop better disease management practices and develop cultivars with enhanced tuber quality and productivity. As a result of our study, we emphasize the need to address ToLCNDV-potato infection in potato production systems to ensure crop health, maximize yields, and produce high-quality tubers. Further research may be conducted in order to determine the mechanisms underlying these adverse effects as well as how they can be mitigated in order to improve the health and productivity of crops.

## Figures and Tables

**Table 1:** Plant height, dry matter content in potato tuber, and stem diameter in control and ToLCNDV infected potato tubers of Kufri Pukhraj and Kufri Bahar varieties.

		Kufri Pukhraj		Kufri Bahar	
S.N.	Parameters	Control	To LCNDV	Control	ToLCNDV
1.	Plant height (cm)	81.90 ± 0.88 ^a^	70.92 ± 1.19 ^b^	67.23 ± 0.34 ^a^	61.24 ± 0.58 ^b^
2.	Dry matter of potato tuber(g)	14.93 ± 0.35 ^a^	11.69 ± 0.32 ^b^	22.93 ± 0.36 ^a^	18.67 ± 0.33 ^b^
3.	Stem diameter (cm)	0.75 ± 0.01 ^a^	0.62 ± 0.01 ^b^	0.62 ± 0.01 ^b^	0.74 ± 0.01 ^a^

Statistical significance of the difference between the means across the variety. All data are represented as mean values ± SEM of three biological replicates. Values with the same superscript in a row are not significantly different (*p* > 0.05) according to Tukey’s multiple comparison test.

## Data Availability

All data generated or analysed during this study are included in this published article and its supplementary information supplementary files.

## Supplementary Materials

The following supporting information can be downloaded at: https://www.mdpi.com/article/10.3390/antiox12071447/s1. Table S1: ANOVA table for interaction between varieties (Kufri Pukhraj and Kufri Bahar) and treatment (healthy control and ToLCNDV infection)for parameters viz., plant height, dry matter of potato tuber, stem diameter, Chl *a*, Chl *b*, total Chl, carotenoid, proline content, MDA content, RWC, relative electric conductivity, starch, sucorse, frucotse, glucose, β-amylase. α-amylase, phosphorylase, catalase, peroxidase, ascorbate oxidase and superoxide dismutase activity. Table S2: Tukey multiple comparison test for plant height of Kufri Pukraj and Kufri Bahar in control (healthy) and ToLCNDV infection plants. Table S3: Tukey multiple comparison test for dry matter of Kufri Pukraj and Kufri Bahar in control (healthy) and ToLCNDV infection plants. Table S4: Tukey multiple comparison test for stem diameter of Kufri Pukraj and Kufri Bahar in control (healthy) and ToLCNDV infection plants. Table S5: Tukey multiple comparison test for Chl a of Kufri Pukraj and Kufri Bahar in control (healthy) and ToLCNDV infection plants. Table S6: Tukey multiple comparison test for Chl b of Kufri Pukraj and Kufri Bahar in control (healthy) and ToLCNDV infection plants. Table S7: Tukey multiple comparison test for total Chl of Kufri Pukraj and Kufri Bahar in control (healthy) and ToLCNDV infection plants. Table S8: Tukey multiple comparison test for carotenoid content of Kufri Pukraj and Kufri Bahar in control (healthy) and ToLCNDV infection plants. Table S9: Tukey multiple comparison test for proline content of Kufri Pukraj and Kufri Bahar in control (healthy) and ToLCNDV infection plants. Table S10: Tukey multiple comparison test for MDA content of Kufri Pukraj and Kufri Bahar in control (healthy) and ToLCNDV infection plants. Table S11: Tukey multiple comparison test for RWC of Kufri Pukraj and Kufri Bahar in control (healthy) and ToLCNDV infection plants. Table S12: Tukey multiple comparison test for relative electric conductivity of Kufri Pukraj and Kufri Bahar in control (healthy) and ToLCNDV infection plants. Table S13: Tukey multiple comparison test for starch content of Kufri Pukraj and Kufri Bahar in control (healthy) and ToLCNDV infection plants. Table S14: Tukey multiple comparison test for sucrose content of Kufri Pukraj and Kufri Bahar in control (healthy) and ToLCNDV infection plants. Table S15: Tukey multiple comparison test for fructose content of Kufri Pukraj and Kufri Bahar in control (healthy) and ToLCNDV infection plants. Table S16: Tukey multiple comparison test for glucose content of Kufri Pukraj and Kufri Bahar in control (healthy) and ToLCNDV infection plants. Table S17: Tukey multiple comparison test for β-amylase of Kufri Pukraj and Kufri Bahar in control (healthy) and ToLCNDV infection plants. Table S18: Tukey multiple comparison test for α-amylase of Kufri Pukraj and Kufri Bahar in control (healthy) and ToLCNDV infection plants. Table S19: Tukey multiple comparison test for phosphorylase of Kufri Pukraj and Kufri Bahar in control (healthy) and ToLCNDV infection plants. Table S20: Tukey multiple comparison test for catalase activity of Kufri Pukraj and Kufri Bahar in control (healthy) and ToLCNDV infection plants. Table S21: Tukey multiple comparison test for peroxidase activity of Kufri Pukraj and Kufri Bahar in control (healthy) and ToLCNDV infection plants. Table S22: Tukey multiple comparison test for ascorbate peroxidase activity of Kufri Pukraj and Kufri Bahar in control (healthy) and ToLCNDV infection plants. Table S23: Tukey multiple comparison test for superoxide dismutase activity of Kufri Pukraj and Kufri Bahar in control (healthy) and ToLCNDV infection plants.

## References

[1] Raymundo R., Asseng S., Robertson R., Petsakos A., Hoogenboom G., Quiroz R., Hareau G., Wolf J. (2018). Climate Change Impact on Global Potato Production. Eur. J. Agron..

[2] Lal M.K., Tiwari R.K., Kumar R., Naga K.C., Kumar A., Singh B., Raigond P., Dutt S., Chourasia K.N., Kumar D. (2021). Effect of Potato Apical Leaf Curl Disease on Glycemic Index and Resistant Starch of Potato (*Solanum tuberosum* L.) Tubers. Food Chem..

[3] Huseynova I.M., Mirzayeva S.M., Sultanova N.F., Aliyeva D.R., Mustafayev N.S., Aliyev J.A. (2018). Virus-Induced Changes in Photosynthetic Parameters and Peroxidase Isoenzyme Contents in Tomato (*Solanum lycopersicum* L.) Plants. Photosynthetica.

[4] Kumar R., Tiwari R.K., Sundaresha S., Kaundal P., Raigond B. (2022). Potato Viruses and Their Management. Sustainable Management of Potato Pests and Diseases.

[5] Kumar R., Tiwari R.K., Jeevalatha A., Siddappa S., Shah M.A., Sharma S., Sagar V., Kumar M., Chakrabarti S.K. (2021). Potato Apical Leaf Curl Disease: Current Status and Perspectives on a Disease Caused by Tomato Leaf Curl New Delhi Virus. J. Plant Dis. Prot..

[6] Usharani K.S., Surendranath B., Paul-Khurana S.M., Garg I.D., Malathi V.G. (2004). Potato Leaf Curl—A New Disease of Potato in Northern India Caused by a Strain of Tomato Leaf Curl New Delhi Virus. Plant Pathol..

[7] Jeevalatha A., Siddappa S., Kumar A., Kaundal P., Guleria A., Sharma S., Nagesh M., Singh B.P. (2017). An Insight into Differentially Regulated Genes in Resistant and Susceptible Genotypes of Potato in Response to Tomato Leaf Curl New Delhi Virus-[Potato] Infection. Virus Res..

[8] Liu R.H. (2013). Health-Promoting Components of Fruits and Vegetables in the Diet. Adv. Nutr..

[9] Kapinga R., Ndunguru J., Mulokozi G., Tumwegamire S. (2009). Impact of Common Sweetpotato Viruses on Total Carotenoids and Root Yields of an Orange-Fleshed Sweetpotato in Tanzania. Sci. Hortic..

[10] Garg I.D., Khurana S.M.P., Kumar S., Lakra B.S. (2001). Association of a Geminivirus with Potato Apical Leaf Curl in India and Its Immuno-Electron Microscopic Detection. J. Indian Potato Assoc..

[11] Alvarez V.H., Cahyadi J., Xu D., Saldaña M.D.A. (2014). Optimization of Phytochemicals Production from Potato Peel Using Subcritical Water: Experimental and Dynamic Modeling. J. Supercrit. Fluids.

[12] Sun H., Fan J., Tian Z., Ma L., Meng Y., Yang Z., Zeng X., Liu X., Kang L., Nan X. (2022). Effects of Treatment Methods on the Formation of Resistant Starch in Purple Sweet Potato. Food Chem..

[13] Lin J.S., Wang G.X. (2002). Doubled CO_2_ could improve the drought tolerance better in sensitive cultivars than in tolerant cultivars in spring wheat. Plant Sci..

[14] Jeevalatha A., Singh B.P., Kaundal P., Kumar R., Raigond B. (2014). RCA-PCR: A Robust Technique for the Detection of Tomato Leaf Curl New Delhi Virus-Potato at Ultra Low Virus Titre. Potato J..

[15] Kumar R., Kaundal P., Arjunan J., Sharma S., Chakrabarti S.K. (2020). Development of a Visual Detection Method for Potato Virus S by Reverse Transcription Loop-Mediated Isothermal Amplification. 3 Biotech.

[16] Zhao B., Liu Q., Wang B., Yuan F. (2021). Roles of Phytohormones and Their Signaling Pathways in Leaf Development and Stress Responses. J. Agric. Food Chem..

[17] Ben Rejeb I., Pastor V., Mauch-Mani B. (2014). Plant Responses to Simultaneous Biotic and Abiotic Stress: Molecular Mechanisms. Plants.

[18] Deja-Sikora E., Werner K., Hrynkiewicz K. (2023). AMF Species Do Matter: Rhizophagus Irregularis and Funneliformis Mosseae Affect Healthy and PVY-Infected *Solanum tuberosum* L. in a Different Way. Front. Microbiol..

[19] Király L., Albert R., Zsemberi O., Schwarczinger I., Hafez Y.M., Künstler A. (2021). Reactive Oxygen Species Contribute to Symptomless, Extreme Resistance to Potato Virus X in Tobacco. Phytopathology.

[20] Kolychikhina M.S., Beloshapkina O.O., Phiri C. (2021). Change in Potato Productivity under the Impact of Viral Diseases. IOP Conf. Ser. Earth Environ. Sci..

[21] Hiscox J.D., Israelstam G.F. (1979). A method for the extraction of chlorophyll from leaf tissue without maceration. Can. J. Bot..

[22] Heath R.L., Packer L. (1968). Photoperoxidation in Isolated Chloroplasts: I. Kinetics and Stoichiometry of Fatty Acid Peroxidation. Arch. Biochem. Biophys..

[23] Steup M., Latzko E. (1979). Intracellular Localization of Phosphorylases in Spinach and Pea Leaves. Planta.

[24] Spychalla J.P., Desborough S.L. (1990). Superoxide dismutase, catalase, and α-tocopherol content of stored potato tubers. Plant Physiol..

[25] Moriones E., Praveen S., Chakraborty S. (2017). Tomato Leaf Curl New Delhi Virus: An Emerging Virus Complex Threatening Vegetable and Fiber Crops. Viruses.

[26] Zhang H., Hou J., Liu J., Zhang J., Song B., Xie C. (2017). The Roles of Starch Metabolic Pathways in the Cold-Induced Sweetening Process in Potatoes. Starch-Stärke.

[27] Flores-Castellanos J., Fettke J. (2023). The Plastidial Glucan Phosphorylase Affects the Maltooligosaccharide Metabolism in Parenchyma Cells of Potato (*Solanum tuberosum* L.) Tuber Discs. Plant Cell Physiol..

[28] Zhang K., Lu H., Wan C., Tang D., Zhao Y., Luo K., Li S., Wang J. (2020). The Spread and Transmission of Sweet Potato Virus Disease (SPVD) and Its Effect on the Gene Expression Profile in Sweet Potato. Plants.

[29] Liu Y., Liu Y., Spetz C., Li L., Wang X. (2020). Comparative Transcriptome Analysis in *Triticum aestivum* Infecting Wheat Dwarf Virus Reveals the Effects of Viral Infection on Phytohormone and Photosynthesis Metabolism Pathways. Phytopathol. Res..

[30] Ratnadass A., Fernandes P., Avelino J., Habib R. (2012). Plant Species Diversity for Sustainable Management of Crop Pests and Diseases in Agroecosystems: A Review. Agron. Sustain. Dev..

[31] Li J.W., Chen H.Y., Li J., Zhang Z., Blystad D.R., Wang Q.C. (2018). Growth, Microtuber Production and Physiological Metabolism in Virus-Free and Virus-Infected Potato In Vitro Plantlets Grown under NaCl-Induced Salt Stress. Eur. J. Plant Pathol..

[32] Zhang S.-h., Xu X.-f., Sun Y.-m., Zhang J.-l., Li C.-z. (2018). Influence of Drought Hardening on the Resistance Physiology of Potato Seedlings under Drought Stress. J. Integr. Agric..

[33] Baebler Š., Stare K., Kovač M., Blejec A., Prezelj N., Stare T., Kogovšek P., Pompe-Novak M., Rosahl S., Ravnikar M. (2011). Dynamics of Responses in Compatible Potato—Potato Virus y Interaction Are Modulated by Salicylic Acid. PLoS ONE.

[34] Jyothsna P., Haq Q.M.I., Singh P., Sumiya K.V., Praveen S., Rawat R., Briddon R.W., Malathi V.G. (2013). Infection of Tomato Leaf Curl New Delhi Virus (ToLCNDV), a Bipartite Begomovirus with Betasatellites, Results in Enhanced Level of Helper Virus Components and Antagonistic Interaction between DNA B and Betasatellites. Appl. Microbiol. Biotechnol..

[35] Shalitin D., Wolf S. (2000). Cucumber Mosaic Virus Infection Affects Sugar Transport in Melon Plants. Plant Physiol..

[36] Yan S.L., Lehrer A.T., Hajirezaei M.R., Springer A., Komor E. (2008). Modulation of Carbohydrate Metabolism and Chloroplast Structure in Sugarcane Leaves Which Were Infected by Sugarcane Yellow Leaf Virus (SCYLV). Physiol. Mol. Plant Pathol..

[37] Herbers K., Takahata Y., Melzer M., Mock H.P., Hajirezaei M., Sonnewald U. (2000). Regulation of Carbohydrate Partitioning during the Interaction of Potato Virus Y with Tobacco. Mol. Plant Pathol..

[38] Gupta U.P., Srivastava M., Gupta U. (2010). Influence of Soybean Mosaic Virus Infection on Carbohydrate Content in Nodule of Soybean (*Glycine max* L. Merr.). Int. J. Virol..

[39] Kulakova A.V., Efremov G.I., Shchennikova A.V., Kochieva E.Z. (2022). Dependence of the Content of Starch and Reducing sugars on the Level of Expression of the Genes of β-Amylases and StBAM9 and the Amylase Inhibitor StAI during Long-Term Low-Temperature Storage of Potato Tubers. Vavilov J. Genet. Breed..

[40] Orzechowski S., Sitnicka D., Grabowska A., Compart J., Fettke J., Zdunek-Zastocka E. (2021). Effect of Short-Term Cold Treatment on Carbohydrate Metabolism in Potato Leaves. Int. J. Mol. Sci..

[41] Sharma P., Jha A.B., Dubey R.S., Pessarakli M. (2012). Reactive Oxygen Species, Oxidative Damage, and Antioxidative Defense Mechanism in Plants under Stressful Conditions. J. Bot..

[42] Sharma N., Muthamilarasan M., Dulani P., Prasad M. (2021). Genomic Dissection of ROS Detoxifying Enzyme Encoding Genes for Their Role in Antioxidative Defense Mechanism against Tomato Leaf Curl New Delhi Virus Infection in Tomato. Genomics.

