# Supplementary material for: Investigating the Interplay between Tomato Leaf Curl New Delhi Virus Infection, Starch Metabolism and Antioxidant Defence System in Potato (Solanum tuberosum L.)
Source: Antioxidants (Basel). 2023 Jul 18;12(7):1447. doi: 10.3390/antiox12071447 (PMC10376058; doi:10.3390/antiox12071447)
Supplement: Supplementary file 1 [file antioxidants-12-01447-s001.zip › antioxidants-2497918-supplementary.pdf]

Table S1: ANOVA table for interaction between varieties (Kufri Pukhraj and Kufri Bahar) and treatment (healthy control and ToLCNDV infection)for parameters viz., plant height, dry matter of potato tuber, stem diameter, Chl *a*, Chl *b*, total Chl, carotenoid, proline content, MDA content, RWC, relative electric conductivity, starch, sucorse, frucotse, glucose, β-amylase. α-amylase, phosphorylase, catalase, peroxidase, ascorbate oxidase and superoxide dismutase activity.

S.N.	Parameters	Varieties	Treatment	V × T
1.	Plant height	ns	***	*
2.	Dry matter of potato tuber	***	***	NS
3.	Stem diameter	***	***	*
4.	Chl a	***	***	NS
5.	Chl b	***	***	NS
6.	Total chl	***	***	NS
7.	Carotenoid	***	***	NS
8.	Proline content	***	***	***
9.	MDA content	***	***	**
10.	Leaf relative water content	***	***	*
11.	Relative electric conductivity	***	***	*
12.	Starch content	***	***	**
13.	Sucrose cotnent	*	***	NS
14.	Fructose content	ns	**	***
15.	Glucose content	*	***	***
16.	β-amylase	ns	***	***
17.	α-amylase	ns	***	***
18.	Phosphorylase	***	***	NS
19.	Catalase activity	**	***	NS
20.	Peroxidase activity	***	***	NS
21.	Ascorbate peroxidase acitvity	***	***	NS
22.	Superoxide dismutase	***	***	***

ns: Not Significant ($p > 0.05$), *significant at $p < 0.05$, **significant at $p < 0.01$, *** significant at $p < 0.001$.

Table S2: Tukey multiple comparison test for plant height of Kufri Pukraj and Kufri Bahar in control (healthy) and ToLCNDV infection plants

Tukey's Multiple Comparisons Tests	Significant?	Summary	Adjusted *p* Value
Kufri Pukhraj:Control vs. Kufri Pukhraj:ToLCNDV	Yes	***	<0.001
Kufri Pukhraj:Control vs. Kufri Bahar:Control	Yes	***	<0.001
Kufri Pukhraj:Control vs. Kufri Bahar:ToLCNDV	Yes	***	<0.001
Kufri Pukhraj:ToLCNDV vs. Kufri Bahar:Control	No	ns	0.074
Kufri Pukhraj:ToLCNDV vs. Kufri Bahar:ToLCNDV	Yes	***	<0.001
Kufri Bahar:Control vs. Kufri Bahar:ToLCNDV	Yes	***	<0.001

ns: Not Significant ($p > 0.05$), *** significant at $p < 0.001$.

Table S3: Tukey multiple comparison test for dry matter of Kufri Pukraj and Kufri Bahar in control (healthy) and ToLCNDV infection plants

Tukey's Multiple Comparisons Tests	Significant?	Summary	Adjusted *p* Value
Kufri Pukhraj:Control vs. Kufri Pukhraj:ToLCNDV	Yes	***	<0.001
Kufri Pukhraj:Control vs. Kufri Bahar:Control	Yes	***	<0.001
Kufri Pukhraj:Control vs. Kufri Bahar:ToLCNDV	Yes	***	<0.001
Kufri Pukhraj:ToLCNDV vs. Kufri Bahar:Control	Yes	***	<0.001
Kufri Pukhraj:ToLCNDV vs. Kufri Bahar:ToLCNDV	Yes	***	<0.001
Kufri Bahar:Control vs. Kufri Bahar:ToLCNDV	Yes	***	<0.001

*** significant at $p < 0.001$.

Table S4: Tukey multiple comparison test for stem diameter of Kufri Pukraj and Kufri Bahar in control (healthy) and ToLCNDV infection plants

Tukey's Multiple Comparisons Tests	Significant?	Summary	Adjusted *p* Value
Kufri Pukhraj:Control vs. Kufri Pukhraj:ToLCNDV	Yes	***	<0.001
Kufri Pukhraj:Control vs. Kufri Bahar:Control	Yes	*	0.016
Kufri Pukhraj:Control vs. Kufri Bahar:ToLCNDV	No	ns	0.905
Kufri Pukhraj:ToLCNDV vs. Kufri Bahar:Control	Yes	***	<0.001
Kufri Pukhraj:ToLCNDV vs. Kufri Bahar:ToLCNDV	Yes	***	<0.001
Kufri Bahar:Control vs. Kufri Bahar:ToLCNDV	Yes	**	0.007

ns: Not Significant ($p > 0.05$), * significant at $p < 0.05$, **significant at $p < 0.01$, *** significant at $p < 0.001$.

Table S5: Tukey multiple comparison test for Chl a of Kufri Pukraj and Kufri Bahar in control (healthy) and ToLCNDV infection plants

Tukey's Multiple Comparisons Tests	Significant?	Summary	Adjusted *p* Value
Kufri Pukhraj:Control vs. Kufri Pukhraj:ToLCNDV	Yes	***	<0.001
Kufri Pukhraj:Control vs. Kufri Bahar:Control	Yes	*	0.033
Kufri Pukhraj:Control vs. Kufri Bahar:ToLCNDV	Yes	**	0.008
Kufri Pukhraj:ToLCNDV vs. Kufri Bahar:Control	Yes	***	<0.001
Kufri Pukhraj:ToLCNDV vs. Kufri Bahar:ToLCNDV	Yes	*	0.038
Kufri Bahar:Control vs. Kufri Bahar:ToLCNDV	Yes	***	<0.001

* significant at $p < 0.05$, **significant at $p < 0.01$, *** significant at $p < 0.001$.

Table S6: Tukey multiple comparison test for Chl b of Kufri Pukraj and Kufri Bahar in control (healthy) and ToLCNDV infection plants

Tukey's Multiple Comparisons Tests	Significant?	Summary	Adjusted *p* Value
Kufri Pukhraj:Control vs. Kufri Pukhraj:ToLCNDV	Yes	***	<0.001
Kufri Pukhraj:Control vs. Kufri Bahar:Control	Yes	**	0.002
Kufri Pukhraj:Control vs. Kufri Bahar:ToLCNDV	No	ns	0.099
Kufri Pukhraj:ToLCNDV vs. Kufri Bahar:Control	Yes	***	<0.001
Kufri Pukhraj:ToLCNDV vs. Kufri Bahar:ToLCNDV	Yes	***	<0.001
Kufri Bahar:Control vs. Kufri Bahar:ToLCNDV	Yes	***	<0.001

ns: Not Significant ($p > 0.05$), **significant at $p < 0.01$, *** significant at $p < 0.001$.

Table S7: Tukey multiple comparison test for total Chl of Kufri Pukraj and Kufri Bahar in control (healthy) and ToLCNDV infection plants

Tukey's Multiple Comparisons Tests	Significant?	Summary	Adjusted *p* Value
Kufri Pukhraj:Control vs. Kufri Pukhraj:ToLCNDV	Yes	***	<0.001
Kufri Pukhraj:Control vs. Kufri Bahar:Control	Yes	**	0.007
Kufri Pukhraj:Control vs. Kufri Bahar:ToLCNDV	Yes	**	0.005
Kufri Pukhraj:ToLCNDV vs. Kufri Bahar:Control	Yes	***	<0.001
Kufri Pukhraj:ToLCNDV vs. Kufri Bahar:ToLCNDV	Yes	**	0.005
Kufri Bahar:Control vs. Kufri Bahar:ToLCNDV	Yes	***	<0.001

significant at $p < 0.01$, * significant at $p < 0.001$.

Table S8: Tukey multiple comparison test for carotenoid content of Kufri Pukraj and Kufri Bahar in control (healthy) and ToLCNDV infection plants

Tukey's Multiple Comparisons Tests	Significant?	Summary	Adjusted *p* Value
Kufri Pukhraj:Control vs. Kufri Pukhraj:ToLCNDV	Yes	**	.003
Kufri Pukhraj:Control vs. Kufri Bahar:Control	Yes	***	<.001
Kufri Pukhraj:Control vs. Kufri Bahar:ToLCNDV	Yes	**	.004
Kufri Pukhraj:ToLCNDV vs. Kufri Bahar:Control	Yes	***	<.001
Kufri Pukhraj:ToLCNDV vs. Kufri Bahar:ToLCNDV	Yes	***	<.001
Kufri Bahar:Control vs. Kufri Bahar:ToLCNDV	Yes	***	<.001

significant at $p < 0.01$, * significant at $p < 0.001$.

Table S9: Tukey multiple comparison test for proline content of Kufri Pukraj and Kufri Bahar in control (healthy) and ToLCNDV infection plants

Tukey's Multiple Comparisons Tests	Significant?	Summary	Adjusted *p* Value
Kufri Pukhraj:Control vs. Kufri Pukhraj:ToLCNDV	Yes	*	0.02
Kufri Pukhraj:Control vs. Kufri Bahar:Control	Yes	***	<0.001
Kufri Pukhraj:Control vs. Kufri Bahar:ToLCNDV	Yes	**	0.001
Kufri Pukhraj:ToLCNDV vs. Kufri Bahar:Control	Yes	***	<0.001
Kufri Pukhraj:ToLCNDV vs. Kufri Bahar:ToLCNDV	No	ns	0.18
Kufri Bahar:Control vs. Kufri Bahar:ToLCNDV	Yes	***	<0.001

ns: Not Significant ($p > 0.05$), * significant at $p < 0.05$, **significant at $p < 0.01$, *** significant at $p < 0.001$.

Table S10: Tukey multiple comparison test for MDA content of Kufri Pukraj and Kufri Bahar in control (healthy) and ToLCNDV infection plants

Tukey's Multiple Comparisons Tests	Significant?	Summary	Adjusted *p* Value
Kufri Pukhraj:Control vs. Kufri Pukhraj:ToLCNDV	Yes	***	<0.001
Kufri Pukhraj:Control vs. Kufri Bahar:Control	Yes	**	0.007
Kufri Pukhraj:Control vs. Kufri Bahar:ToLCNDV	Yes	*	0.015
Kufri Pukhraj:ToLCNDV vs. Kufri Bahar:Control	Yes	***	<0.001
Kufri Pukhraj:ToLCNDV vs. Kufri Bahar:ToLCNDV	Yes	***	<0.001
Kufri Bahar:Control vs. Kufri Bahar:ToLCNDV	Yes	***	<0.001

* significant at $p < 0.05$, **significant at $p < 0.01$, *** significant at $p < 0.001$.

Table S11: Tukey multiple comparison test for RWC of Kufri Pukraj and Kufri Bahar in control (healthy) and ToLCNDV infection plants

Tukey's Multiple Comparisons Tests	Significant?	Summary	Adjusted *p* Value
Kufri Pukhraj:Control vs. Kufri Pukhraj:ToLCNDV	Yes	***	<0.001
Kufri Pukhraj:Control vs. Kufri Bahar:Control	No	ns	0.288
Kufri Pukhraj:Control vs. Kufri Bahar:ToLCNDV	Yes	***	<0.001
Kufri Pukhraj:ToLCNDV vs. Kufri Bahar:Control	Yes	***	<0.001
Kufri Pukhraj:ToLCNDV vs. Kufri Bahar:ToLCNDV	Yes	***	<0.001
Kufri Bahar:Control vs. Kufri Bahar:ToLCNDV	Yes	***	<0.001

ns: Not Significant ($p > 0.05$), *** significant at $p < 0.001$.

Table S12: Tukey multiple comparison test for relative electric conductivity of Kufri Pukraj and Kufri Bahar in control (healthy) and ToLCNDV infection plants

Tukey's Multiple Comparisons Tests	Significant?	Summary	Adjusted *p* Value
Kufri Pukhraj:Control vs. Kufri Pukhraj:ToLCNDV	Yes	***	<0.001
Kufri Pukhraj:Control vs. Kufri Bahar:Control	Yes	*	0.019
Kufri Pukhraj:Control vs. Kufri Bahar:ToLCNDV	Yes	***	<0.001
Kufri Pukhraj:ToLCNDV vs. Kufri Bahar:Control	Yes	***	<0.001
Kufri Pukhraj:ToLCNDV vs. Kufri Bahar:ToLCNDV	Yes	***	<0.001
Kufri Bahar:Control vs. Kufri Bahar:ToLCNDV	Yes	***	<0.001

* significant at $p < 0.05$, *** significant at $p < 0.001$.

Table S13: Tukey multiple comparison test for starch content of Kufri Pukraj and Kufri Bahar in control (healthy) and ToLCNDV infection plants

Tukey's Multiple Comparisons Tests	Significant?	Summary	Adjusted *p* Value
Kufri Pukhraj:Control vs. Kufri Pukhraj:ToLCNDV	Yes	***	<0.001
Kufri Pukhraj:Control vs. Kufri Bahar:Control	Yes	***	<0.001
Kufri Pukhraj:Control vs. Kufri Bahar:ToLCNDV	Yes	***	<0.001
Kufri Pukhraj:ToLCNDV vs. Kufri Bahar:Control	Yes	***	<0.001
Kufri Pukhraj:ToLCNDV vs. Kufri Bahar:ToLCNDV	No	ns	0.897
Kufri Bahar:Control vs. Kufri Bahar:ToLCNDV	Yes	***	<0.001

ns: Not Significant ($p > 0.05$), *** significant at $p < 0.001$.

Table S14: Tukey multiple comparison test for sucrose content of Kufri Pukraj and Kufri Bahar in control (healthy) and ToLCNDV infection plants

**Tukey's Multiple Comparisons Tests**	**Significant?**	**Summary**	**Adjusted *p* Value**
Kufri Pukhraj:Control vs. Kufri Pukhraj:ToLCNDV	Yes	**	0.002
Kufri Pukhraj:Control vs. Kufri Bahar:Control	No	ns	0.703
Kufri Pukhraj:Control vs. Kufri Bahar:ToLCNDV	No	ns	0.131
Kufri Pukhraj:ToLCNDV vs. Kufri Bahar:Control	Yes	***	<0.001
Kufri Pukhraj:ToLCNDV vs. Kufri Bahar:ToLCNDV	No	ns	0.058
Kufri Bahar:Control vs. Kufri Bahar:ToLCNDV	Yes	*	0.028

ns: Not Significant ($p > 0.05$), * significant at $p < 0.05$, **significant at $p < 0.01$, *** significant at $p < 0.001$.

Table S15: Tukey multiple comparison test for fructose content of Kufri Pukraj and Kufri Bahar in control (healthy) and ToLCNDV infection plants

**Tukey's Multiple Comparisons Tests**	**Significant?**	**Summary**	**Adjusted *p* Value**
Kufri Pukhraj:Control vs. Kufri Pukhraj:ToLCNDV	Yes	***	<0.001
Kufri Pukhraj:Control vs. Kufri Bahar:Control	Yes	**	0.006
Kufri Pukhraj:Control vs. Kufri Bahar:ToLCNDV	Yes	***	<0.001
Kufri Pukhraj:ToLCNDV vs. Kufri Bahar:Control	Yes	***	<0.001
Kufri Pukhraj:ToLCNDV vs. Kufri Bahar:ToLCNDV	Yes	**	0.002
Kufri Bahar:Control vs. Kufri Bahar:ToLCNDV	Yes	***	<0.001

significant at $p < 0.01$, * significant at $p < 0.001$.

Table S16: Tukey multiple comparison test for glucose content of Kufri Pukraj and Kufri Bahar in control (healthy) and ToLCNDV infection plants

**Tukey's Multiple Comparisons Tests**	**Significant?**	**Summary**	**Adjusted *p* Value**
Kufri Pukhraj:Control vs. Kufri Pukhraj:ToLCNDV	Yes	***	<0.001
Kufri Pukhraj:Control vs. Kufri Bahar:Control	Yes	**	0.004
Kufri Pukhraj:Control vs. Kufri Bahar:ToLCNDV	Yes	***	<0.001
Kufri Pukhraj:ToLCNDV vs. Kufri Bahar:Control	Yes	***	<0.001
Kufri Pukhraj:ToLCNDV vs. Kufri Bahar:ToLCNDV	Yes	***	<0.001
Kufri Bahar:Control vs. Kufri Bahar:ToLCNDV	Yes	***	<0.001

significant at $p < 0.01$, * significant at $p < 0.001$.

Table S17: Tukey multiple comparison test for β-amylase of Kufri Pukraj and Kufri Bahar in control (healthy) and ToLCNDV infection plants

**Tukey's Multiple Comparisons Tests**	**Significant?**	**Summary**	**Adjusted *p* Value**
Kufri Pukhraj:Control vs. Kufri Pukhraj:ToLCNDV	Yes	***	<0.001
Kufri Pukhraj:Control vs. Kufri Bahar:Control	Yes	**	0.004
Kufri Pukhraj:Control vs. Kufri Bahar:ToLCNDV	Yes	***	<0.001
Kufri Pukhraj:ToLCNDV vs. Kufri Bahar:Control	Yes	***	<0.001
Kufri Pukhraj:ToLCNDV vs. Kufri Bahar:ToLCNDV	Yes	***	<0.001
Kufri Bahar:Control vs. Kufri Bahar:ToLCNDV	Yes	**	0.002

significant at $p < 0.01$, * significant at $p < 0.001$.

Table S18: Tukey multiple comparison test for α-amylase of Kufri Pukraj and Kufri Bahar in control (healthy) and ToLCNDV infection plants

**Tukey's Multiple Comparisons Tests**	**Significant?**	**Summary**	**Adjusted *p* Value**
Kufri Pukhraj:Control vs. Kufri Pukhraj:ToLCNDV	Yes	***	<0.001
Kufri Pukhraj:Control vs. Kufri Bahar:Control	Yes	***	<0.001
Kufri Pukhraj:Control vs. Kufri Bahar:ToLCNDV	Yes	***	<0.001
Kufri Pukhraj:ToLCNDV vs. Kufri Bahar:Control	Yes	***	<0.001
Kufri Pukhraj:ToLCNDV vs. Kufri Bahar:ToLCNDV	Yes	*	0.010
Kufri Bahar:Control vs. Kufri Bahar:ToLCNDV	Yes	***	<0.001

* significant at $p < 0.05$, *** significant at $p < 0.001$.

Table S19: Tukey multiple comparison test for phosphorylase of Kufri Pukraj and Kufri Bahar in control (healthy) and ToLCNDV infection plants

**Tukey's Multiple Comparisons Tests**	**Significant?**	**Summary**	**Adjusted *p* Value**
Kufri Pukhraj:Control vs. Kufri Pukhraj:ToLCNDV	Yes	***	<0.001
Kufri Pukhraj:Control vs. Kufri Bahar:Control	Yes	**	0.005
Kufri Pukhraj:Control vs. Kufri Bahar:ToLCNDV	Yes	***	<0.001
Kufri Pukhraj:ToLCNDV vs. Kufri Bahar:Control	Yes	**	0.009
Kufri Pukhraj:ToLCNDV vs. Kufri Bahar:ToLCNDV	Yes	*	0.014
Kufri Bahar:Control vs. Kufri Bahar:ToLCNDV	Yes	***	<0.001

* significant at $p < 0.05$, **significant at $p < 0.01$, *** significant at $p < 0.001$.

Table S20: Tukey multiple comparison test for catalase activity of Kufri Pukraj and Kufri Bahar in control (healthy) and ToLCNDV infection plants

**Tukey's Multiple Comparisons Tests**	**Significant?**	**Summary**	**Adjusted *p* Value**
Kufri Pukhraj:Control vs. Kufri Pukhraj:ToLCNDV	Yes	*	0.021
Kufri Pukhraj:Control vs. Kufri Bahar:Control	No	ns	0.272
Kufri Pukhraj:Control vs. Kufri Bahar:ToLCNDV	Yes	***	<0.001
Kufri Pukhraj:ToLCNDV vs. Kufri Bahar:Control	No	ns	0.313
Kufri Pukhraj:ToLCNDV vs. Kufri Bahar:ToLCNDV	Yes	*	0.027
Kufri Bahar:Control vs. Kufri Bahar:ToLCNDV	Yes	**	0.003

ns: Not Significant ($p > 0.05$), * significant at $p < 0.05$, **significant at $p < 0.01$, *** significant at $p < 0.001$.

Table S21: Tukey multiple comparison test for peroxidase activity of Kufri Pukraj and Kufri Bahar in control (healthy) and ToLCNDV infection plants

**Tukey's Multiple Comparisons Tests**	**Significant?**	**Summary**	**Adjusted *p* Value**
Kufri Pukhraj:Control vs. Kufri Pukhraj:ToLCNDV	Yes	***	<0.001
Kufri Pukhraj:Control vs. Kufri Bahar:Control	Yes	**	0.002
Kufri Pukhraj:Control vs. Kufri Bahar:ToLCNDV	Yes	***	<0.001
Kufri Pukhraj:ToLCNDV vs. Kufri Bahar:Control	Yes	***	<0.001
Kufri Pukhraj:ToLCNDV vs. Kufri Bahar:ToLCNDV	Yes	**	0.005
Kufri Bahar:Control vs. Kufri Bahar:ToLCNDV	Yes	***	<0.001

significant at $p < 0.01$, * significant at $p < 0.001$.

Table S22: Tukey multiple comparison test for ascorbate peroxidase activity of Kufri Pukraj and Kufri Bahar in control (healthy) and ToLCNDV infection plants

**Tukey's Multiple Comparisons Tests**	**Significant?**	**Summary**	**Adjusted *p* Value**
Kufri Pukhraj:Control vs. Kufri Pukhraj:ToLCNDV	Yes	***	<0.001
Kufri Pukhraj:Control vs. Kufri Bahar:Control	Yes	***	<0.001
Kufri Pukhraj:Control vs. Kufri Bahar:ToLCNDV	Yes	***	<0.001
Kufri Pukhraj:ToLCNDV vs. Kufri Bahar:Control	Yes	**	0.007
Kufri Pukhraj:ToLCNDV vs. Kufri Bahar:ToLCNDV	Yes	***	<0.001
Kufri Bahar:Control vs. Kufri Bahar:ToLCNDV	Yes	***	<0.001

significant at $p < 0.01$, * significant at $p < 0.001$.

Table S23: Tukey multiple comparison test for superoxide dismutase activity of Kufri Pukraj and Kufri Bahar in control (healthy) and ToLCNDV infection plants

Tukey's Multiple Comparisons Tests	Significant?	Summary	Adjusted *p* Value
Kufri Pukhraj:Control vs. Kufri Pukhraj:ToLCNDV	Yes	***	<0.001
Kufri Pukhraj:Control vs. Kufri Bahar:Control	Yes	***	<0.001
Kufri Pukhraj:Control vs. Kufri Bahar:ToLCNDV	Yes	***	<0.001
Kufri Pukhraj:ToLCNDV vs. Kufri Bahar:Control	No	ns	0.494
Kufri Pukhraj:ToLCNDV vs. Kufri Bahar:ToLCNDV	Yes	***	<0.001
Kufri Bahar:Control vs. Kufri Bahar:ToLCNDV	Yes	***	<0.001

ns: Not Significant ($p > 0.05$), *** significant at $p < 0.001$.
